# *Cis*-2-dodecenoic acid signal modulates virulence of *Pseudomonas aeruginosa* through interference with quorum sensing systems and T3SS

**DOI:** 10.1186/1471-2180-13-231

**Published:** 2013-10-18

**Authors:** Yinyue Deng, Calvin Boon, Shaohua Chen, Amy Lim, Lian-Hui Zhang

**Affiliations:** 1 Institute of Molecular and Cell Biology, Proteos, 61 Biopolis Drive, Singapore, 138673, Singapore; 2 College of Natural Resources and Environment, South China Agricultural University, Guangzhou, People's Republic of China

## Abstract

**Background:**

*Cis*-2-dodecenoic acid (BDSF) is well known for its important functions in intraspecies signaling in *Burkholderia cenocepacia*. Previous work has also established an important role of BDSF in interspecies and inter-kingdom communications. It was identified that BDSF modulates virulence of *Pseudomonas aeruginosa*. However, how BDSF interferes with virulence of *P. aeruginosa* is still not clear.

**Results:**

We report here that BDSF mediates the cross-talk between *B. cenocepacia* and *P. aeruginosa* through interference with quorum sensing (QS) systems and type III secretion system (T3SS) of *P. aeruginosa*. Bioassay results revealed that exogenous addition of BDSF not only reduced the transcriptional expression of the regulator encoding gene of QS systems, i.e., *lasR*, *pqsR*, and *rhlR*, but also simultaneously decreased the production of QS signals including 3-oxo-C12-HSL, *Pseudomonas* quinolone signal (PQS) and C4-HSL, consequently resulting in the down-regulation of biofilm formation and virulence factor production of *P. aeruginosa*. Furthermore, BDSF and some of its derivatives are also capable of inhibiting T3SS of *P. aeruginosa* at a micromolar level. Treatment with BDSF obviously reduced the virulence of *P. aeruginosa* in both HeLa cell and zebrafish infection models.

**Conclusions:**

These results depict that BDSF modulates virulence of *P. aeruginosa* through interference with QS systems and T3SS.

## Background

Many microorganisms utilize species-specific small signal molecules to coordinate a range of important activities, including virulence factor production, antibiotics biosynthesis and biofilm. This cell-cell communication mechanism is known as quorum sensing (QS) [1-4]. As a ubiquitous environmental organism which infects various animals, plants, and insects, *Pseudomonas aeruginosa* is also a major source of opportunistic infections in both immunocompromised individuals and cystic fibrosis patients [5,6]. It has evolved at least three types of quorum sensing (QS) systems, i.e., *las*, *pqs* and *rhl*, which are implicated in regulation of several aspects of pathogenesis, including virulence factor production, biofilm development, and antimicrobial resistance [7]. Besides the QS systems, most clinical isolates of *P. aeruginosa* also use type III secretion system (T3SS) to evade phagocytosis and facilitate infection [8-13]. T3SS is an important virulence determinant which is conserved in many animal and plant pathogens, including *Salmonella* spp., *Shigella flexneri*, *Yersinia* spp., *Escherichia coli*, and *Chlamydia* spp. [14,15]. Although previous study already revealed that QS controls T3SS in some bacterial species, the relationship between QS systems and T3SS in *P. aeruginosa* is still not clearly determined [16,17].

There is a new form of microbe-microbe antagonism interaction designated as signal interference [18]. This type of antagonism acts not by killing, but instead by interfering with the signal-mediated gene expression of the competitors [19,20]. It has been found that some microorganisms could boost their competitive strength through interfering with QS signaling of their competitors [18]. This interference mechanism has been employed to develop novel drugs as the antagonists of signaling systems of bacterial pathogens. For example, some compounds have been identified or synthesized to act as the antagonists of QS systems of *P. aeruginosa*, including *N*-acyl cyclopentylamides, Furanone derivatives, Garlic, Malyngolide, Iberin, Protoanemonin, Norbgugaine and Caffeine [21-28]. Besides the signal interference with QS system, interfering with T3SS system has also been shown as an effective method to treat the bacterial pathogens [29-31]. Given the fact that the anti-QS or anti-T3SS compounds display a significantly inhibitory activity on the virulence of *P. aeruginosa*, suggesting that interference on QS systems or/and T3SS can be specifically utilized as favorable therapeutic methods on *P. aeruginosa* infection [32-34].

As a new type of QS signal, the diffusible signal factor (DSF) has been demonstrated to play an important role to control the biological functions such as biofilm formation, motility, virulence and antibiotic resistance [3,4]. Among DSF-family signals, *Cis*-2-dodecenoic acid (BDSF) was originally identified in *Burkholderia cenocepacia* to be involved in regulation of biofilm formation, virulence and motility in *B. cenocepacia*[35-41]. Furthermore, besides the significance on intraspecies signaling, BDSF also appears its critical importance for maintenance of ecology through interspecies and inter-kingdom communication [35,37,42]. Exogenous addition of BDSF from *B. cenocepacia* restored the biofilm dispersal and virulence factor production of *Xanthomonas campestris* pv. *campestris* DSF-deficient mutants. It was also revealed that *Candida albicans* germ tube formation was strongly inhibited by exogenous addition of physiological relevant level of BDSF [35]. Moreover, BDSF from *B. cenocepacia* and *Stenotrophomonas maltophilia* was found to be involved in modulation of virulence, antibiotic resistance and persistence of *P. aeruginosa* in the cystic fibrosis airway [42]. In combination, these results have well established the role of BDSF play not only in regulation of a range of biological functions through intraspecies signaling, but also in microbial ecology through interspecies and cross-kingdom communication. In this study, we investigate the role of BDSF in the inter-species communication between *B. cenocepacia* and *P. aeruginosa*; and discover the inhibitory effect of BDSF on QS systems and T3SS of *P. aeruginosa*.

## Results

### BDSF interferes with QS systems of *P. aeruginosa*

There are at least two AHL-dependent QS systems in *P. aeruginosa*, *las* and *rhl* systems, which control the expression of numerous genes [34,43,44]. It was found that the two QS systems are in a hierarchy where *las* system is dominant over *rhl* system [45,46]. Moreover, it was also identified that LasR was required for the production of PQS, which plays a positive effect on *rhl* system. The three QS systems are interconnected with *las* system at the top of the QS cascade, which controls *rhl* and *pqs* systems [47,48]. Recently, it was revealed that *pqs* and *rhl* systems are under the positive regulation of *iqs* system (It was named for its role in integrating the QS network in *P. aeruginosa*), which is tightly controlled by *las* system in *P. aeruginosa*[49].

**Figure 1:**
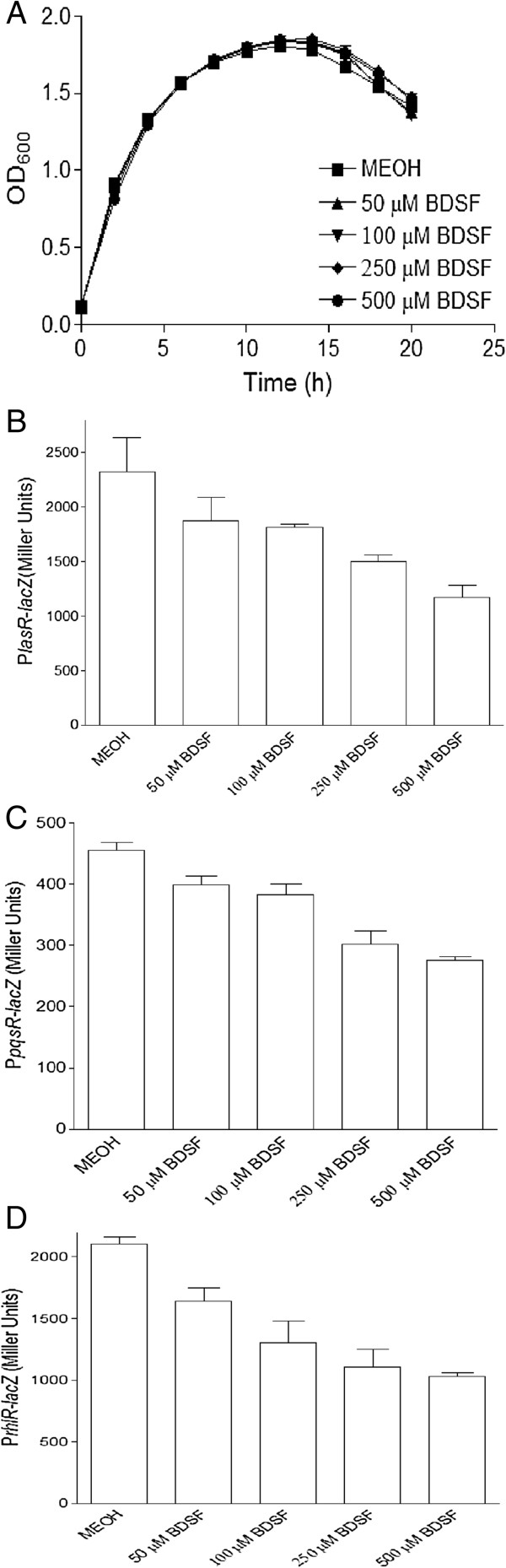
**Influence of BDSF on QS systems of *P. aeruginosa*.** Effect of BDSF on the growth rate of *P. aeruginosa* PA14 **(A)**, and on the transcriptional expression of *lasR***(B)**, *pqsR***(C)**, and *rhlR***(D)**, as determined by using corresponding promoter-*lacZ* fusion reporter strains. The data are the means of three repeats and error bars indicate the standard deviations.

It was found that there is a regulatory interaction between the different type QS systems, BDSF and AHL systems in *B. cenocepacia*[39,40]. Disruption of both BDSF synthase and receptor caused a substantial down-regulation of AHL signals production and AHL synthase gene expression in *B. cenocepacia*. Given the fact that *B. cenocepacia* and *P. aeruginosa* share the same niche in cystic fibrosis patent, we then hypothesized that BDSF may play a regulatory role in the QS systems of *P. aeruginosa*. To determine the influences of BDSF on the QS systems of *P. aeruginosa*, the promoter-lacZ fusion reporters were constructed to test the transcriptional expression of the regulator encoding gene of QS systems, i.e., *lasR*, *pqsR* and *rhlR*, in the absence and presence of BDSF. It was shown that exogenous addition of BDSF did not obviously affect the bacterial growth of *P. aeruginosa* PA14 (Figure 1A). But the transcriptional expressions of *lasR*, *pqsR* and *rhlR* were repressed by treatment with BDSF in a dosage-dependent manner. Addition of 0.25 mM BDSF resulted in 38%, 35% and 48% reduction of the expression of *lasR*, *pqsR* and *rhlR*, respectively (Figure 1B-D). To further investigate BDSF effect on the QS signal production of *P. aeruginosa* PA14, production of 3-oxo-C12-HSL, PQS, and C4-HSL were tested in the absence and presence of BDSF. Consistently, it was revealed that treatment with BDSF decreased the production of 3-oxo-C12-HSL, PQS and C4-HSL. As shown in Additional file 1: Figure S1, addition of BDSF slightly decreased the production of 3-oxo-C12-HSL; while obviously inhibited the production of PQS and C4-HSL.

### BDSF inhibits biofilm formation and virulence factor production of *P. aeruginosa*

**Figure 2:**
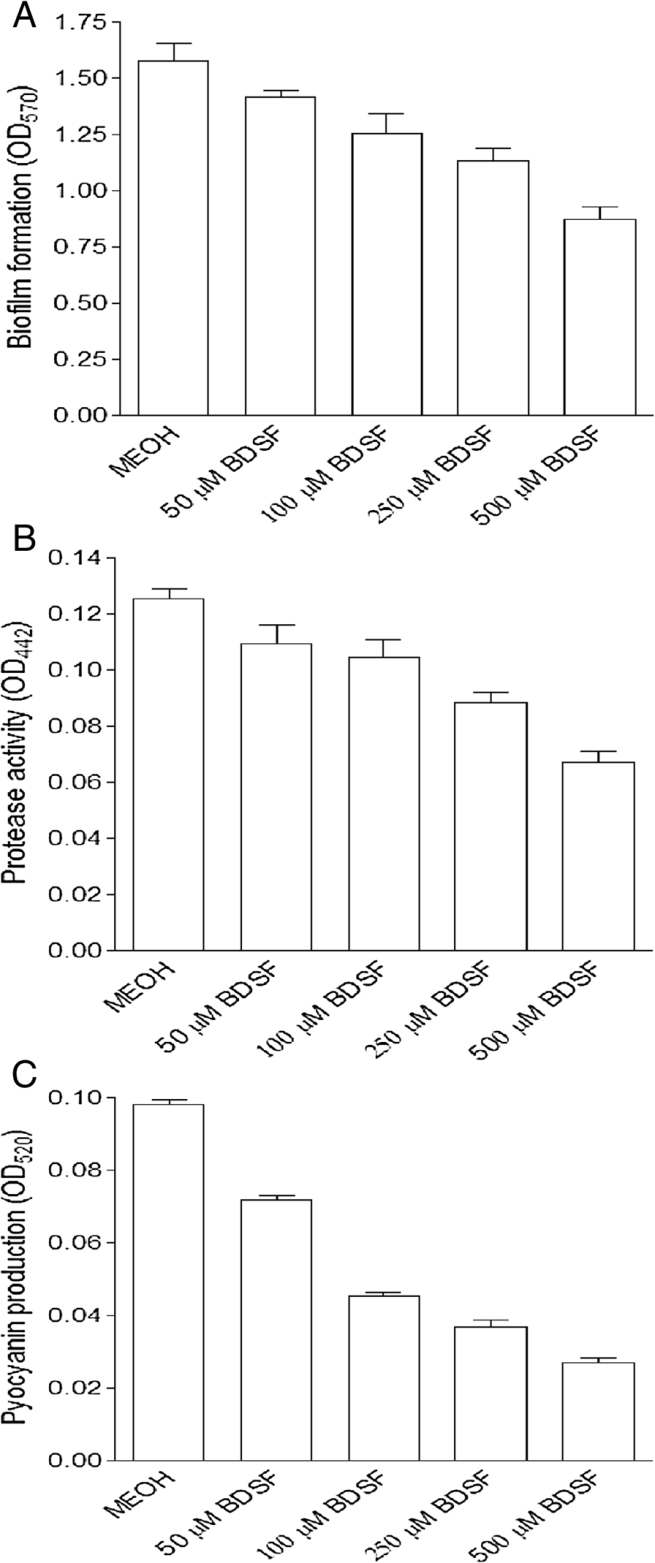
**Inhibitory effect of BDSF on biofilm formation (A), production of extracellular protease (B) and pyocyanin (C) of *P. aeruginosa *PA14.** The data are the means of three repeats and error bars indicate the standard deviations.

It is well known that QS systems in *P. aeruginosa* modulate biofilm development, virulence factor production, and antimicrobial resistance [7]. Linking with the finding of the inhibitory activity of BDSF on the QS systems, we then continued to determine the effect of BDSF on the biofilm formation and virulence factor production. As shown in Figure 1A, exogenous addition of BDSF showed no obvious effect on the growth rate of *P. aeruginosa* PA14; while it remarkably decreased the biofilm formation. Addition of 0.05, 0.1, 0.25 and 0.5 mM BDSF reduced the biofilm formation by 10.2%, 20.2%, 27.9%, and 44.%, respectively (Figure 2A).

*P. aeruginosa* usually utilizes exoenzyme to induce its pathogenesis [50,51]. To study the effect of BDSF on the production of exoproteases, which are important virulence factors of *P. aeruginosa*, BDSF was added to the growth medium of *P. aeruginosa* PA14 to test its effect on the secretion of proteases in the supernatants. Results demonstrated that addition of BDSF inhibited the production of proteases, treatment with 0.25 mM BDSF caused 30% reduction of the protease activity (Figure 2B). The reduction was increased to 50% when 0.5 mM BDSF was supplemented (Figure 2B).

During the growth process, it was observed that BDSF inhibited the pigment accumulation of *P. aeruginosa* PA14 in the medium. *P. aeruginosa* produces a number of colored secondary metabolities; one of them is pyocyanin, which is a virulence factor [52,53]. To determine whether BDSF affects this virulence factor production, we measured pyocyanin accumulation in LB medium in the absence and presence of BDSF. As shown in Figure 2C, addition of BDSF to the bacterial medium substantially reduced the production of pyocyanin. Compared with the control, addition of 0.1 and 0.5 mM BDSF reduced the pyocyanin production by about 55% and 70%, respectively (Figure 2C).

It was reported that DSF from *S. maltophilia* influences biofilm formation and polymyxin tolerance in *P. aeruginosa* through the sensor kinase PA1396 [54]. To test whether PA1396 is also the sensor of BDSF in *P. aeruginosa*, we then measured the protease activity and pyocyanin production of *PA1396* deletion mutant in the absence and presence of BDSF. However, our results showed that disruption of *PA1396* was not able to diminish the inhibitory effect of BDSF on the virulence factor production (Additional file 1: Figure S2), suggesting that PA1396 is not BDSF sensor kinase in *P. aeruginosa*.

### Exogenous addition of BDSF represses T3SS of *P. aeruginosa*

**Figure 3:**
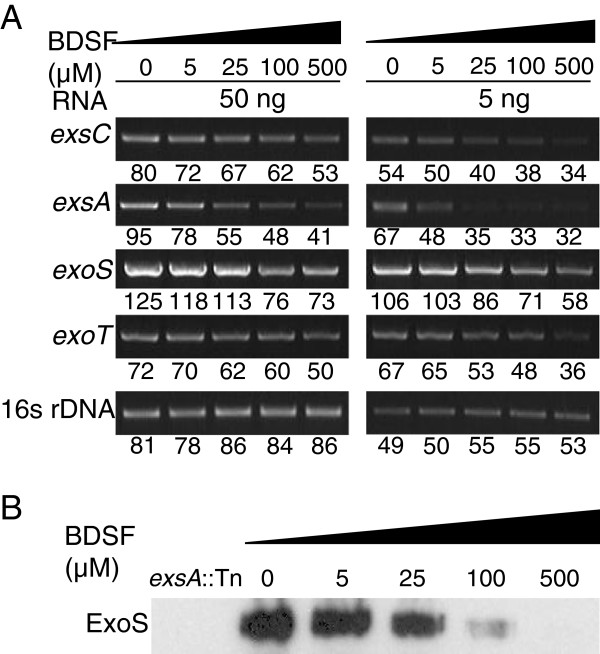
**Exogenous addition of BDSF caused the reduction of the transcriptional expression of T3SS master regulators and effectors, as determined by using RT-PCR analysis (A), and of secretion of ExoS determined by western blotting analysis (B).** Bacteria were grown in LB medium to an OD_600_ of 1.5, supplemented with 5 mM NTA and BDSF at a series of final concentrations as indicated. For each RNA sample, two dilutions (5, 50 ng) were used as templates for RT-PCT reaction. For the western blotting analysis, the extra-cellular proteins in supernatants were collected by trichloroacetic acid precipitation and separated by 10% SDS-PAGE. The proteins were transferred onto nitrocellulose membrane and blotted with anti-ExoS antibody.

Besides the QS systems, T3SS is also an important virulence determinant in *P. aeruginosa*. We firstly studied the effect of BDSF on T3SS of *P. aeruginosa* by using semi-quantitative RT-PCR. At the panel of 5 ng RNA, results showed that addition of 100 μM BDSF to *P. aeruginosa* led to about 30% and 50% reduction in the signal density of *exsC* and *exsA*, which are the master regulators and positively control the expression of T3SS effectors genes in *P. aeruginosa* (Figure 3A) [13,55,56]. We then continued to measure the effect of BDSF on T3SS effectors. Semi-quantitative RT-PCR analysis showed that treatment of *P. aeruginosa* PA14 with 100 μM BDSF caused about 39% and 17% reduction in transcripts levels of *exoS* and *exoT* at the panel of 50 ng RNA, respectively (Figure 3A). Furthermore, western blotting assay was used to analyze the effect of BDSF on the secreted ExoS in supernatant. As shown in Figure 3B, addition of 100 μM BDSF significantly reduced the amount of ExoS secreted in supernatant. When the final concentration of BDSF was increased to 500 μM, there was almost no detectable protein band of ExoS.

It was previously reported that a long-chain fatty acid (LCFA) negatively modulates the expression of type III *exsCEBA* operon in *P. aeruginosa* through the sensor PsrA [57]. To investigate whether BDSF shares the same signaling pathway as LCFA, we then further measured the effect of BDSF on T3SS in *P. aeruginosa psrA* mutant. Unexpectedly, deletion of *psrA* displayed no effect on the inhibitory activity of BDSF on T3SS of *P. aeruginosa*, suggesting that BDSF possibly acts through another sensor different from that of the LCFA molecule (Additional file 1: Figure S3).

### BDSF inhibits T3SS of *P. aeruginosa* at its physiological relevant level

**Figure 4:**
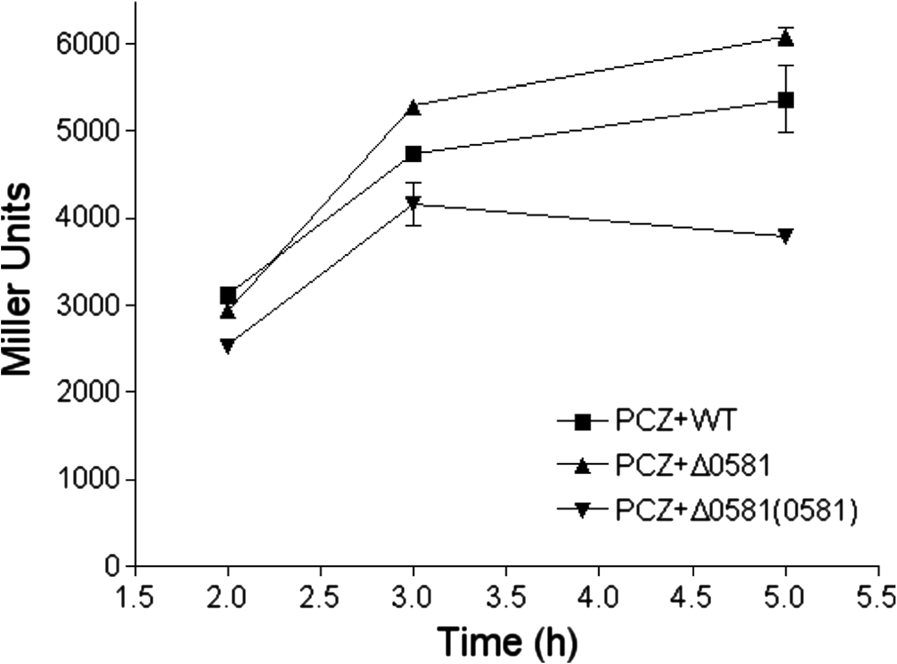
**Inhibitory effect of BDSF on T3SS of *P. aeruginosa *at its physiological relevant level, as determined by using P*exsCEBA-lacZ *fusion reporter strain.** Bacteria were grown in the mixture of LB broth with cell-free supernatants of *B. cenocepacia* J2315 wild-type (■), Δ0581 (▲), Δ0581(0581) (▼) in a ratio of 1:1 (v/v), supplemented with 5 mM NTA. The data are the means of three repeats and error bars indicate the standard deviations.

To determine whether BDSF affects T3SS gene expression in *P. aeruginosa* PA14 at its physiological relevant level, T3SS reporter P*exsCEBA-lacZ* was used to test the T3SS gene expression at transcriptional level. *B. cenocepacia* cells were firstly grown in Luria Bertani (LB) broth to an OD_600_ of 3.5; and cultures were centrifuged to collect the supernatants, which were then filtered with 0.2 μm syringe filter to remove the remaining cells. T3SS reporter strain was inoculated in the mixture of the supernatant of *B. cenocepacia* strains and LB broth at a ratio of 1:1 (v/v), with supplementation of 5 mM nitrilotiracetic acid (NTA). The bioassay result showed that the reporter strain displayed the greatest activity when it was cultured in the supernatant of BDSF-deficient mutant; while the supernatants of both the wild-type strain and over-expression complementary strain showed a remarkable inhibition on the T3SS gene expression at the time points examined (Figure 4).

### Many BDSF derivatives show inhibitory activity on T3SS of *P. aeruginosa*

**Figure 5:**
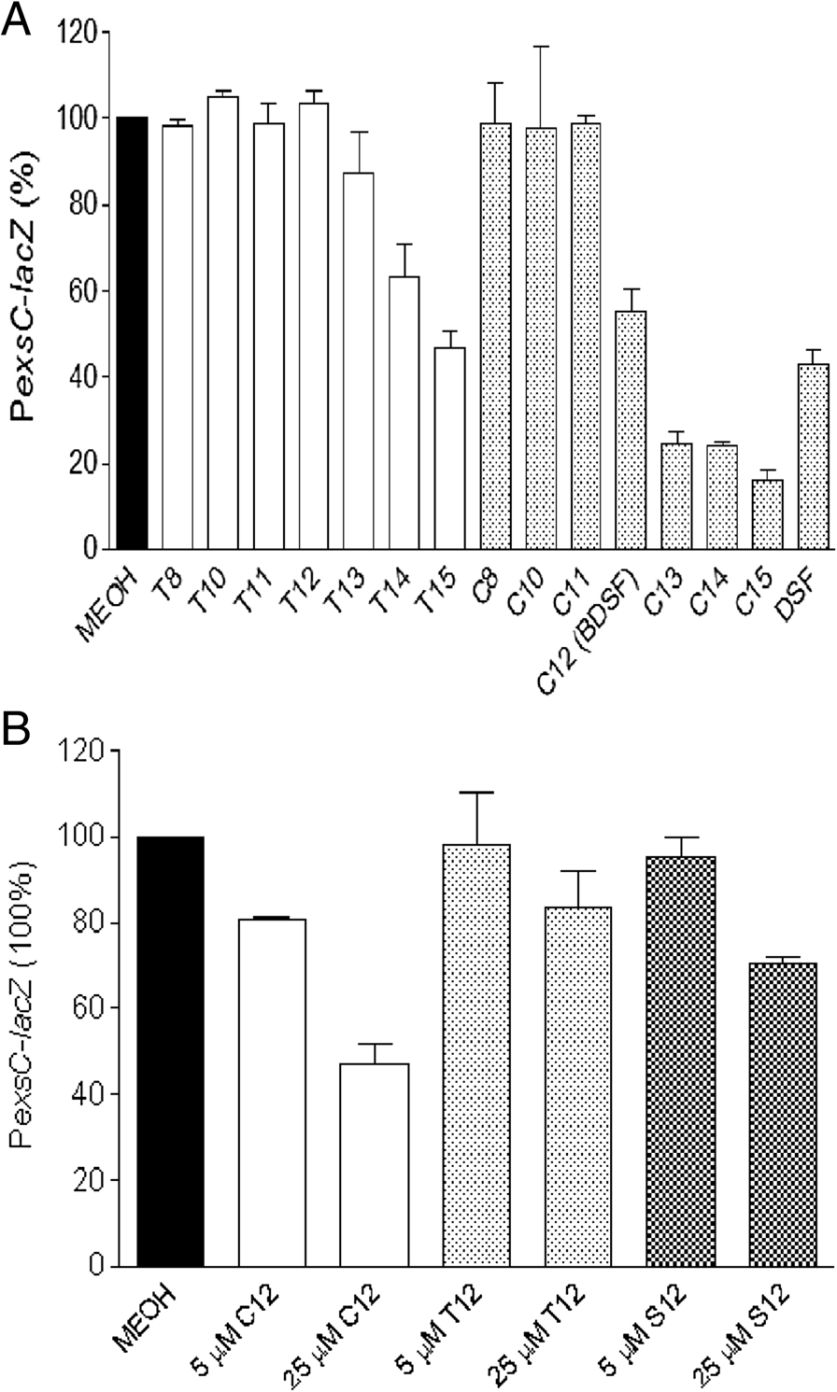
**The inhibitory activity on T3SS of *P. aeruginosa *PA14 is conserved in BDSF derivatives. (A)** Effect of exogenous addition of 10 μM BDSF and its derivatives on T3SS genes expression, as determined by using P*exsCEBA-lacZ* fusion reporter strain. “T” means the fatty acids with *trans* configuration; while “**C**” means the fatty acids with *cis* configuration; number represents the carbon chain length. Their structures were listed in Table S1. **(B)** BDSF (C12) showed the strongest activity than its saturated isomer (S12) and *trans*-isomer (T12). Bacteria were grown in LB medium supplemented with 5 mM NTA to an OD_600_ of ~1.5. The data are the means of three repeats and error bars indicate the standard deviations.

It was revealed that structural features of fatty acids molecules may contribute to their biological activity [4,37,58]. To investigate whether BDSF derivatives share the inhibitory activity on T3SS of *P. aeruginosa*, and study the interaction between structural features and inhibitory activities of DSF-family molecules on T3SS, a series of BDSF derivatives with different structures (Additional file 1: Table S1) were applied to test their effect on the transcriptional expression of *exsCEBA*. The T3SS reporter strain, P*exsCEBA-lacZ*, was refreshed and cultured in LB medium supplemented with 5 mM NTA. BDSF and its derivatives were added at a final concentration of 10 μM. Beta-gal activity was measured when the cultures reached an OD_600_ of ~1.5. Bioassays results revealed that α,β-unsaturated fatty acids with chain length more than twelve showed a significant inhibition on T3SS gene expression (Figure 5). Moreover, it was indicated that configuration of BDSF derivatives contributes to their inhibitory activity; and *cis*-conformational fatty acids showed the strongest inhibitory activity, followed by their saturated isomers and *trans*-isomers (Figure 5B). Additionally, methyl group substitution and chain length could also affect the inhibitory activity (Figure 5A).

### BDSF attenuates the virulence of *P. aeruginosa* in both *in vitro* and *in vivo* assays

**Figure 6:**
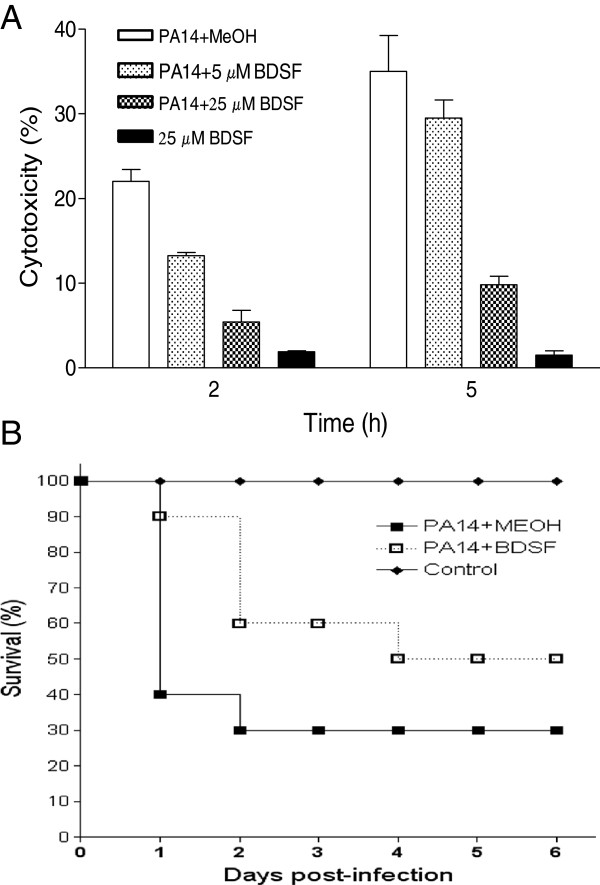
**BDSF attenuates the virulence of *P. aeruginosa *PA14 in both *in vitro *and *in vivo *model.** Cytotoxicity was assayed by monitoring LDH released by the HeLa cells infected with a MOI of about 50. Experiments were performed with DMEM medium supplemented with BDSF. The data are the means of three repeats and error bars indicate the standard deviations **(A)**. Zebra-fish model was assayed by testing the survival rate of the fish after infection with *P. aeruginosa* PA14, without (■) and with (□) treatment of BDSF. PBS containing relevant BDSF was injected in the same way as for a mock control (♦). The experiment was repeated three times, and a representative set of data is shown **(B)**.

To determine the effect of BDSF on the virulence of *P. aeruginosa* PA14, HeLa cells were firstly used as the *in vitro* model. Results indicated that exogenous addition of BDSF significantly decreased the cytotoxicity of *P. aeruginosa* PA14 to HeLa cell. For 2 hours inoculation, the cytotoxicity was reduced by 41% and 75% with treatment of 5 μM and 25 μM BDSF, respectively (Figure 6A). While for 5 hours inoculation, the reductions were 16% and 73%, respectively (Figure 6A).

Zebrafish was reported to be a good infection model for the test of T3SS-mediated virulence of *P. aeruginosa*[59]. We then analyzed the effect of BDSF on the virulence of *P. aeruginosa* PA14 by infecting 6-month-old zebrafish (*Danio rerio*), which was used as an infection model for *B. cenocepacia*[36]. As shown in Figure 6B, treatment with 100 μM BDSF significantly reduced the virulence of *P. aeruginosa* PA14 in zebrafish infection model. While 60% of the fish infected with *P. aeruginosa* PA14 died within 24 hours post-inoculation, the *P. aeruginosa* PA14 treated with BDSF was much less virulent and only 10% of the infected fish died (Figure 6B).

## Discussion

This study demonstrated that besides acting as an intraspecies signal, BDSF can also mediate inter-species communication between *B. cenocepacia* and *P. aeruginosa* through inhibiting on QS systems and T3SS of *P. aeruginosa*. Previous studies already found that there is a cross-talk between *B. cepacia* and *P. aeruginosa*, which is modulated by AHL QS signal from *P. aeruginosa*[60,61]. It was reported that addition of cell-free exoproducts of PAO1 to *B. cepacia* markedly enhanced its production of siderophore, lipase and protease; while addition of the supernatants of PAO1 with attenuated production of AHL only had slight effect on the production of these virulence factors [60]. Moreover, AHL from PAO1 can also induce the biofilm formation of *B. cenocepacia* H111, demonstrating that AHL signal plays an important role in the cross-talk between the two bacterial species, which usually share the same niche in cystic fibrosis patients. Recently, the critical role of BDSF in the interspecies communication between *B. cenocepacia* and *P. aeruginosa* was also well established by the finding that BDSF modulates virulence, antibiotic resistance and persistence of *P. aeruginosa* in the cystic fibrosis airway [42]. Our findings not only further confirmed the BDSF role in the complicated interaction between *B. cenocepacia* and *P. aeruginosa*, but also depicted how BDSF modulates the virulence of *P. aeruginosa*.

In *P. aeruginosa*, there are at least three QS systems, i.e., *las*, *pqs* and *rhl*, consisting of a hierarchy regulatory network. These QS systems regulate a broad range of genes important for the metabolism and virulence of *P. aeruginosa*. Inhibition on these QS systems will finally attenuate the virulence factor production and virulence of *P. aeruginosa*. Our study showed that addition of BDSF caused the decreased expression of *lasR*, which is the regulator of *las* system (Figure 1B). Consistently with the dominant role of *las* system over *pqs* and *rhl* systems, addition of BDSF also obviously inhibited *pqs* and *rhl* systems (Figure 1C-D, Additional file 1: Figure S1), suggesting that the inhibitory activity of BDSF on the QS systems of *P. aeruginosa* may initial from the inhibition on *las* system. Recently, a DSF sensor, PA1396 was identified in *P. aeruginosa*[54]. To determine whether BDSF and DSF share the same sensor in *P. aeruginosa*, we then tested the BDSF effect on the virulence factor production mediated by QS systems in PA1396 deletion mutant. However, it was revealed that PA1396 is not BDSF sensor kinase, as disruption of PA1396 could not diminish the inhibitory effect of BDSF on the virulence factor production (Additional file 1: Figure S2). How does BDSF interfere with QS systems in *P. aeruginosa* still needs the further investigation.

T3SS is a key virulence determinant in a wide range of animal and plant pathogens and plays diverse roles in host-pathogen interactions. In *P. aeruginosa*, the transcriptional expression of effector genes of T3SS is coordinated by ExsA encoded by the *exsCEBA* operon. Our results showed that BDSF inhibited the expression of *exsCEBA*, which was confirmed by the results of semi-quantitative RT-PCR analysis and western blotting analysis (Figure 3). It was recently reported that PsrA serves as a sensor for a long chain fatty acid (LCFA) to negatively modulate the expression of T3SS in *P. aeruginosa*[57]. However, exogenous addition of BDSF still showed a similar inhibition pattern on *psrA* deletion mutant, suggesting that BDSF may control T3SS through another sensor (Additional file 1: Figure S3). Moreover, the fact that T3SS is more sensitive to the exogenous addition of BDSF than the QS systems indicates that BDSF may interfere with QS systems and T3SS of *P. aeruginosa* through two independent signaling pathways. Besides the inhibitory effect on the QS systems and T3SS, our microarray analysis data suggests that BDSF also affects many other genes in *P. aeruginosa* (Data not shown). It was found that treatment with 250 μM of BDSF up-regulated 66 genes more than 2-fold, which is 1.38% to the total number of genes in genome. Meanwhile, a total of 120 genes were down-regulated by more than 2-fold (Data not shown). These genes are classified into the functional groups of metabolism, secretion, motility and cell wall, transcription regulation, protection, enzymes and carbon compound catabolism (Data not shown), suggesting that BDSF plays a global effect on *P. aeruginosa*.

Antibiotics have been used for a long time to treat the bacterial infection of *P. aeruginosa*, but resistance to antibiotics could be evolved during clinic treatment. Interestingly, recently studies found that some compounds successfully interfered with *N*-acyl homoserine lactone of *P. aeruginosa* and suppressed bacterial QS in lungs, finally caused accelerated lung bacterial clearance and reduced the severity of lung pathology [32]. Furthermore, some other compounds were identified to be the inhibitors of *P. aeruginosa* T3SS, treatment with these compounds caused the attenuated cytotoxicity of *P. aeruginosa* on mammalian cells [31]. Combined with our findings, it is suggested that interferences on the QS systems and T3SS can be specifically utilized as favorable therapeutic methods on *P. aeruginosa* infection [31,32,34].

## Conclusions

BDSF mediates the cross-talk between *B. cenocepacia* and *P. aeruginosa* by interfering with QS systems and T3SS of *P. aeruginosa*. Biological functions such as biofilm formation and virulence factor production of *P. aeruginosa* were inhibited by exogenous addition of BDSF. Furthermore, BDSF and some of its derivatives are also able to inhibit T3SS of *P. aeruginosa* at a micromolar level. Treatment with BDSF obviously reduced the virulence of *P. aeruginosa* in both *in vitro* and *in vivo* models.

## Methods

### Bacterial strains and growth conditions

The strains used in this work are listed in Table 1. *P. aeruginosa* PA14 strains were maintained in Luria-Bertani (LB) broth at 37°C. For analysis of T3SS, bacteria were grown in LB medium supplemented with the chelating reagent nitrilotiracetic acid (NTA) at a final concentration of 5 mM NTA. The following antibiotics were supplemented when necessary: tetracycline, 100 μg ml^-1^; kanamycin, 50 μg ml^-1^; ampicillin, 200 μg ml^-1^. BDSF and its derivatives were synthesized as described previously [58]. They were added to the medium as indicated.

**Table 1:** Bacterial strains and plasmids used in this study

**Strain or plasmid**	**Phenotypes and/or characteristics**	**Reference or source**
*B. cenocepacia*		
WT	J2315 Wild type strain, Genomovars III of *B. cepacia* complex	ATCC
d0581	BDSF-minus mutant derived from J2315 with *Bcam0581* being deleted	35
d0581(0581)	Mutant d0581 harboring the expression construct pMLS7-0581	35
*P. aeruginosa*		
PA14	Clinical isolate	Laboratory collection
PA14(PCZ)	PA14 harboring the T3SS reporter construct mini-CTX-lacZ with the *exs*CEBA promoter fused to lacZ	This study
PA14 (PlasR-lacZ)	PA14 harboring the reporter construct pME2PlasR-lacZ	This study
PA14 (PpqsR-lacZ)	PA14 harboring the reporter construct pME2PpqsR-lacZ	This study
PA14 (PrhlR-lacZ)	PA14 harboring the reporter construct pME2PrhlR-lacZ	This study
dpsrA (PCZ)	*psrA* deletion mutant harboring the T3SS reporter construct mini-CTX-lacZ with the exsCEBA promoter fused to lacZ	This study
dPA1396	PA1396 deletion mutant derived from PA14	This study
*E.coli*		
S17-1	Res^-^ pro mod^+^ integrated copy of RP4, mob^+^, used for incorporating	Laboratory collection
CV026	The biosensor strain of C4-HSL	Laboratory collection
*Agrobacterium tumefaciens*		
CF11	AHL reporter strain	Lab of Stephen K. Farrand
Plasmid		
PCZ	mini-CTX-lacZ with the *exsCEBA* promoter fused to lacZ	62
PlasR-lacZ	pME2-lacZ containing promoter of *lasR*	This study
PpqsR-lacZ	pME2-lacZ containing promoter of *pqsR*	This study
PrhlR-lacZ	pME2-lacZ containing promoter of *rhlR*	This study

### Bacterial growth analysis

Overnight bacterial cultures grown in LB broth was inoculated in the same medium to an OD_600_ of 0.025 in the absence and presence of BDSF. Three hundred microliters of inoculated culture were grown in each well at 37°C in a low intensity shaking model using the Bioscreen-C Automated Growth Curves Analysis System (OY Growth Curves AB Ltd, Finland).

### Construction of reporter strains and measurement of *β-*galactosidase activity

The promoters of *lasR*, *rhlR* and *pqsR* were amplified using the primer pairs listed in Additional file 1: Table S2 with *Hin*dIII and *Eco*RI restriction sites attached. The resulting products were digested with *Hin*dIII and *Eco*RI, and ligated at the same enzyme sites in the vector pME2-lacZ. These constructs, verified by DNA sequencing, were introduced into *P. aeruginosa* by electroporation. Transconjugants were then selected on LB agar plates supplemented with tetracycline. For the construction of reporter strain of T3SS, the promoter of *exsCEBA* was amplified by PCR using the primer pairs listed in Additional file 1: Table S2, and cloned into the integration vector mini-CTX-lacZ [62]. The construct was introduced into *E. coli* S17-1(λpir) and then integrated into the chromosome of *P. aeruginosa* as described previously [63]. The engineered strain was then selected on the LB agar plates containing tetracycline and used as the T3SS reporter strain. Measurement of *β*-galactosidase activities was assayed following the methods as described previously [64]. Bacteria were grown at 37°C to an OD_600_ of about 1.5, and the cells were harvested to measure the *β*-galactosidase activities.

### Biofilm formation assays

Biofilm formation in 96-well polypropylene microtitre dishes was assayed as followed. Overnight bacterial cultures grown in LB broth was inoculated in the same medium to an OD_600_ of 0.01 in the absence and presence of BDSF signal at different concentrations as indicated. One hundred microliters of inoculated culture were grown in each well at 37°C with shaking at 150 rpm for 18 h. The cultures were removed and 200 μl of 1% crystal violet (w/v) was added. Following staining at room temperature for 15 min, the dye was removed and the wells were rinsed three times with water. For quantification of the attached bacterial cells, the stained wells were decolorized with 200 μl of 95% ethanol. The quantity of crystal violet was determined by measuring the absorbance at 595 nm.

### Proteolytic activity assay

Protease activity was quantified following the previously described method with minor modifications [65]. Briefly, bacteria were cultured at 37°C for about 12 hours. After measuring the optical density at 600 nm, cultures were centrifuged at 13,000 rpm for 5 min and the supernatants were taken out and filtered through a 0.2 μm pore size cellulose-acetate filter. One hundred microliter of supernatants were incubated at 30°C with equal volume of azocasein dissolved in proteolytic buffer B (5 mg/ml) for 30 min. The reaction was stopped by addition of 400 μl of 10% (w/v) TCA buffer. After incubation for 2 min at room temperature, the mixture was centrifuged at 13,000 rpm for 1 min to remove the remaining azocasein. Supernatants were taken out and mixed with 700 μl of 525 mM NaOH. Absorbance of the azopeptide supernatant was measured at the wavelength of 442 nm. Protease activity was obtained after normalization the absorbance against the corresponding cell density.

### Pyocyanin assay

After measuring the absorbance at 600 nm, the supernatants of bacterial cultures were collected for extraction of pyocyanin with the method described previously [66]. Briefly, cultures were centrifuged at 13,000 rpm for 1 min and 1.5 ml supernatants were collected and extracted with double volume chloroform with vigorous shaking at room temperature for 30 min. The solvent phase was transferred to a new tube containing 1 ml of 1 N HCl. The mixture was shaken gently to transfer pyocyanin to aqueous phase. The quantity of pyocyanin was determined by measurement of absorbance at 520 nm and normalization against the cell density.

### RNA extraction and RT-PCR analysis

*P. aeruginosa* was grown in LB medium supplemented with NTA till OD_600_ of 1.5. Total RNA was isolated using the RNeasy mini kit (Qiagen) according to the manufacturer’s instructions. The concentration and purity of RNA were determined by agarose gel electrophoresis and spectrometry. Reverse transcription (RT-PCR) analysis was performed using the One-step RT-PCR kit according to the manufacturer’s instructions (Qiagen).

### Protein isolation and western blotting analysis

Bacteria were cultured in LB medium supplemented with NTA to an OD_600_ of 1.5, 10 ml of each bacterial culture were collected and centrifuged. The supernatants were then filtered with 0.2 μm syringe filter and precipitated with trichloroacetic acid (TCA) at a final concentration of 10%. The precipitates were pelleted by centrifugation, washed twice with acetone, dried, and re-suspended in SDS sampling buffer. The protein samples were denatured by boiling for 5 min and separated by 10% SDS-PAGE. Western blot analysis was performed following the standard protocols [67].

### Cytotoxicity assays in HeLa cell model

BDSF effect on the cytotoxicity of *P. aeruginosa* 14 was assayed by using HeLa cells. HeLa cells were seeded in 24-well tissue culture plates containing Dulbecco’s Modified Eagle Medium (DMEM) and allowed to grow at 37°C in CO_2_ for about 18 hours to obtain 80-90% monolayer confluency (5.0×10^5^ cells/well). Culture supernatants were removed and the monolayer was washed once with PBS buffer. Fresh bacterial cells were diluted in DMEM to a concentration about 5×10^7^ CFU per ml. Thereafter, 0.5 ml of bacteria dilutions in the absence and presence of BDSF were applied to the HeLa cell monolayers at a multiplicity of infection (MOI) about 50. Cytotoxicity was determined by measuring the release of the cytosolic enzyme lactate dehydrogenase (LDH) into supernatants using the cytotoxicity detection kit (Roche).

### Virulence assays using a zebrafish infection model

*P. aeruginosa* PA14 virulence was tested by infecting 6-month-old zebrafish (*Danio rerio*). Firstly, bacterial cultures were grown to an OD_600_ of 1.0, and washed with phosphate-buffered saline (PBS; pH 7.3), then dissolved in PBS buffer to 5.0 × 10^9^ cfu ml^-1^ in the presence of 100 μM BDSF or equal volume methanol, respectively. To this end, 30 μl bacterial cultures were injected intraperitoneally into each fish by using a 1-ml tuberculin syringe attached to a 30.5-gauge Precision Glide needle (Becton Dickinson). The experiment was repeated three times, each time using 10 fish of similar body mass per treatment. Mortality was scored daily, and dead fish was removed immediately.

Zebrafish experiments were conducted in accordance with the guidelines of Institutional Biosafety Committee and Institutional Ethics committee (IBC-IEC) of Institute of Molecular and Cell Biology (IMCB) of Singapore. Animal infection experiments were approved by IBC-ITE of IMCB.

## Competing interest

The authors declare that they have no competing interest.

## Authors’ contributions

Experiments were carried out by YD, BC, SC, AL. Data analysis was finished by YD and LHZ. The study was designed by YD and LHZ, who also drafted the manuscript. All authors read and approved the final manuscript.

## Supplementary Material

Additional file 1: Figure S1 Inhibition of exogenous addition of BDSF on the production of 3-oxo-C12-HSL (A), PQS (B) and C4-HSL (C) of *P. aeruginosa* PA14. **Figure S2.** Inhibitory effect of BDSF on the production of extracellular protease (A) and pyocyanin (B) of PA1396 deletion mutant of *P. aeruginosa* PA14. The data are the means of three repeats and error bars indicate the standard deviations. **Figure S3.** Inhibitory effect of BDSF on T3SS of *psrA* deletion mutant of *P. aeruginosa* PA14, as determined by using P*exsCEBA-lacZ* fusion reporter strain. The data are the means of three repeats and error bars indicate the standard deviations. **Table S1.** Chemical structures of BDSF and its derivatives. **Table S2.** PCR primers used in this study. Click here for file
