# Supplementary material for: Cis-2-dodecenoic acid signal modulates virulence of Pseudomonas aeruginosa through interference with quorum sensing systems and T3SS
Source: BMC Microbiol. 2013 Oct 18;13:231. doi: 10.1186/1471-2180-13-231 (PMC4016476; doi:10.1186/1471-2180-13-231)
Supplement: Additional file 1: Figure S1 — Inhibition of exogenous addition of BDSF on the production of 3-oxo-C12-HSL (A), PQS (B) and C4-HSL (C) of P. aeruginosa PA14. Figure S2. Inhibitory effect of BDSF on the production of extracellular protease (A) and pyocyanin (B) of PA1396 deletion mutant of P. aeruginosa PA14. The data are the means of three repeats and error bars indicate the standard deviations. Figure S3. Inhibitory effect of BDSF on T3SS of psrA deletion mutant of P. aeruginosa PA14, as determined by using PexsCEBA-lacZ fusion reporter strain. The data are the means of three repeats and error bars indicate the standard deviations. Table S1. Chemical structures of BDSF and its derivatives. Table S2. PCR primers used in this study. [file 1471-2180-13-231-S1.docx]

**Additional file 1**

**Deng et al., “***Cis*-2-dodecenoic acid signal modulates virulence of *Pseudomonas aeruginosa* through interference with quorum sensing systems and T3SS**”.**

**Figure S1.** Inhibition of exogenous addition of BDSF on the production of 3-oxo-C12-HSL (A), PQS (B) and C4-HSL (C) of *P. aeruginosa* PA14.

**Figure S2.** Inhibitory effect of BDSF on the production of extracellular protease (A) and pyocyanin (B) of PA1396 deletion mutant of *P. aeruginosa* PA14. The data are the means of three repeats and error bars indicate the standard deviations.

**Figure S3.** Inhibitory effect of BDSF on T3SS of *psrA* deletion mutant of *P. aeruginosa* PA14, as determined by using P*exsCEBA-lacZ* fusion reporter strain. The data are the means of three repeats and error bars indicate the standard deviations.

**Table S1.** Chemical structures of BDSF and its derivatives.

**Table S2.** PCR primers used in this study.

**Experimental methods:**

**PQS assay**

PQS production was assayed following the previous method (Fletcher *et al*., 2007). In brief, overnight starter culture of *P. aeruginosa* was inoculated in LB liquid medium to an OD_600_ of 0.05 and then grown at 37^o^C for about 16 hours to a similar cell density. The culture was centrifuged and 5 ml of supernatants were taken out and added with equal volume of acidified ethyl acetate. After mixing vigorously for 5 min, the top organic layer was collected. The organic solvent was removed by rotary evaporation, and the residue was dissolved in 100 μl methanol as the PQS extract for further analysis.

To prepare the PQS assay, the normal phase silica gel TLC plates (MERCK) were activated by soaking in a 5% (w/v) solution of KH_2_PO_4_ for 30 min, and then dried at 100^o^C for 2 hours. For each sample, 10 μl of extract was spotted onto the TLC plate with synthetic PQS used as a positive control. Then the TLC plate was placed in a developing tank containing a mixture of dichloromethane: methanol (95:5, v/v) as the mobile phase until the solvent front reached about 1-2 cm from the top of the plate. It was visualized using a UV transilluminator at 312 nm. The signal density of PQS was determined using ImageJ (http://rsb.info.nih.gov/ij/).

**3-oxo-C12-HSL and C4-HSL assay**

Bacteria culture supernatants (25 ml) were collected by centrifugation when it grew to an OD_600_ of about 2.2, and extracted with equal volume of the acidified ethyl acetate. The extracts were dried and dissolved in 50 μl methanol. Quantification of 3-oxo-C12-HSL signals was performed using β-galactosidase assay with the aid of the AHL reporter strain CF11 as described previously (Zhang *et al*., 1993). Briefly, the reporter strain was grown in minimal medium at 28^o^C with shaking at 220 rpm overnight. The cultures were inoculated in the same medium supplemented with extracts containing 3-oxo-C12-HSL signals. Bacterial cells were harvested and β-galactosidase activities were measured. *N*-butyryl-L-homoserine lactone (C4-HSL) production was assayed by using its biosensor *Chromobacterium violaceum* strain CV026 (McClean *et al*., 1997). Bioassay plates were prepared by growing CV026 overnight at 28^o^C in LB liquid medium and diluted in the ratio of 1:100 in melted LB agar (42^o^C). The wells about 4-mm diameter were punched in the middle of the plates and 2.5 μl extracts was added to each well. The plates were incubated at 28^o^C overnight. C4-HSL was indicated by the presence of purple halo around the well.

References:

1. Fletcher MP, Diggle SP, Ca´mara M, Williams P: **Biosensor-based assays for PQS, HHQ and related 2-alkyl-4-quinolone quorum sensing signal molecules.** *Nat Protoc* 2007, **2:**1254-1262.
2. Zhang LH, Murphy P, Kerr A, Tate M: **Agrobacterium conjugation and gene regulation by N-acyl-L-homoserine lactones.** *Nature* 1993, **362:**446–447.
3. McClean KH, Winson MK, Fish L, Taylor A, Chhabra SR, Camara M, Daykin M, Lamb JH, Swift S, Bycroft BW, Stewart GS, Williams P: **Quorum sensing and Chromobacterium violaceum: exploitation of violacein production and inhibition for the detection of N-acyl homoserine lactones.** *Microbiology* 1997, **143:**3703–3711.


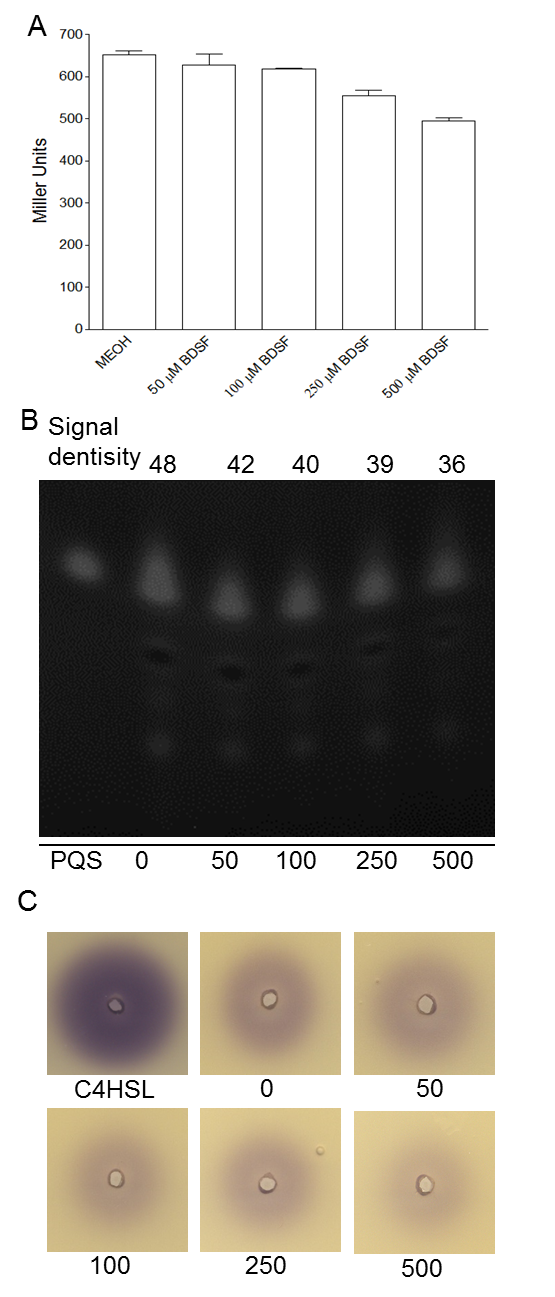


**Figure S1.** Inhibition of exogenous addition of BDSF on the production of 3-oxo-C12-HSL (A), PQS (B) and C4-HSL (C) of *P. aeruginosa* PA14.


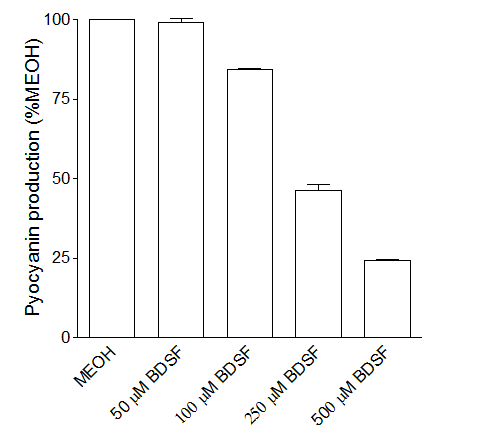

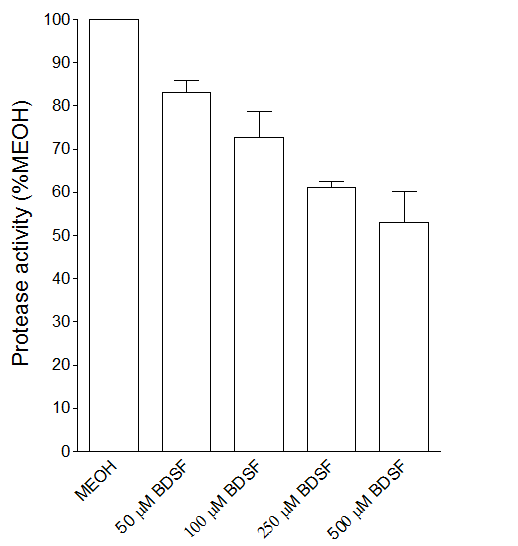


B

A

**Figure S2.** Inhibitory effect of BDSF on the production of extracellular protease (A) and pyocyanin (B) of PA1396 deletion mutant of *P. aeruginosa* PA14. The data are the means of three repeats and error bars indicate the standard deviationws.

**Figure S3.** Inhibitory effect of BDSF on T3SS of *psrA* deletion mutant of *P. aeruginosa* PA14, as determined by using P*exsCEBA-lacZ* fusion reporter strain. The data are the means of three repeats and error bars indicate the standard deviations.

**Table S1.** Chemical structures of BDSF and its derivatives

| **Compound** | **Configuration** | | **Structure** | | **References** | | |  |
| --- | --- | --- | --- | --- | --- | --- | --- | --- |
| T8 | | *trans* |  | | Wang et al., 2004 | | |  |
| T10 | | *trans* |  | | Wang et al., 2004 | | |  |
| T11 | | *trans* |  | | Wang et al., 2004 | | |  |
| T12 | | *trans* |  | | Wang et al., 2004 | | |  |
| T13 | | *trans* |  | | Wang et al., 2004 | | |  |
| T14 | | *trans* |  | | Wang et al., 2004 | | |  |
| T15 | | *trans* |  |  | |  | Wang et al., 2004 | |
| C8 | | *cis* |  |  | |  | Wang et al., 2004 | |
| C10 | | *cis* |  | | Wang et al., 2004 | | |  |
| C11 | | *cis* |  | | Wang et al., 2004 | | |  |
| C12 | | *cis* |   | | Boon et al., 2008 | | |  |
| DSF | | *cis* |  | | Wang et al., 2004 | | |  |
| C13 | | *cis* |  | | This study | | |  |
| C14 | | *cis* |  | | Wang et al., 2004 | | |  |
| C15 | | *cis* |  | | Wang et al.,2004 | | |  |
| S12 | | NT |  | | This study | | |  |

References:

1. Wang LH, He Y, Gao Y, Wu JE, Dong YH, He C, Wang SX, Weng LX, Xu JL, Tay L, Fang RX, Zhang LH : **A bacterial cell-cell communication signal with cross-kingdom structural analogues.** *Mol Microbiol* 2004, **51:**903-912.
2. Boon C, Deng Y, Wang LH, He Y, Xu JL, Fan Y, Pan SQ, Zhang LH: **A novel DSF-like signal from *Burkholderia cenocepacia* interferes with Candida albicans morphological transition.** *ISME J* 2008, **2:**27–36.

**Table S2**. PCR primers used in this study

| **Primer** | **Sequence (5’-3’)** |
| --- | --- |
| *For reporter* |  |
| pC-F | 5’- gctctagacggtgatccagtccttc |
| pC-R | 5’- ggggcgcctcctaaagctc |
| F-pLasR-HindIII | 5’- cgatgggccgacagtgaacc |
| R-pLasR-EcoRI | 5’- ctgcaggatggcgctccactc |
| F-pRhlR-HindIII | 5’- ggtgccgcaggtgctgctg |
| R-pRhlR-EcoRI | 5’- gtggatcggctgcatctcgc |
| F-pqsR-HindIII | 5’- gtgcgtcatagtcgctacacctgaag |
| R-pqsR-EcoRI | 5’- ccgacggaccagctccacg |
| *For RT-PCR analysis* |  |
| exsA-F | 5’- ggcggcgatagctctgggtgaaat |
| exsA-R | 5’- cgccgcggaagctatgtcgtaagt |
| exsC-F | 5’- tggatttaacgagcaaggtcaa |
| exsC-R | 5’- cgagaatctgcgcatacaactg |
| exoS-F | 5’- ctcggccgtcgtgttcaagcagat |
| exoS-R | 5’- ccggggttcagggaggtggaga |
| exoT-F | 5’-caggcgccgctctcccgtcag |
| exoT-R | 5’-ctccgcctccagcccgaagtgc |
